# Corporoplasty: A simplified technique for clitoroplasty

**DOI:** 10.1590/S1677-5538.IBJU.2022.0018

**Published:** 2022-02-22

**Authors:** Ubirajara Barroso, Eliakim Massuqueto, Bruna A. Venturini, Tiago Elias Rosito, Marcelo Villalta, Leidy Paola Casas Grajales

**Affiliations:** 1 Departamento de Urologia Universidade Federal da Bahia Salvador BA Brasil Departamento de Urologia, Universidade Federal da Bahia - UFBA, Salvador, BA, Brasil;; 2 Departamento de Urologia Hospital de Clínicas de Porto Alegre RS Brasil Departamento de Urologia, Hospital de Clínicas de Porto Alegre, RS, Brasil

## Abstract

**Introduction:**

Clitoroplasty constitutes an important step in feminizing surgery for congenital adrenal hyperplasia (CAH) ( 1 ). In this video we present a technique that aims to preserve clitoral sensitivity and engorgement while minimizing the risk of neurovascular lesion.

**Materials and methods:**

We present a video of a three-year-old girl with history of CAH classical form, PRADER-III, who underwent clitoroplasty. After an initial endoscopic evaluation of the urogenital sinus, the clitoris was degloved and a rectangular incision was made on the ventral corpora cavernosa 15mm above the corpora bifurcation and 0.5 mm below the coronal sulcus. The cavernous tissue was partially resected. The upper and lower borders of the rectangular gap were closed by a 5-0 PDS running suture similar to the Mikulicz technique. Next, the edge of the glans was deepithelialized to reduce its size. For improved clitoral positioning, the clitoris was sutured to the pubic fat. From that point onward the procedure followed that of a standard vaginoplasty using the en-bloc technique ( 2 - 4 ). Thus far we have performed this technique in 33 patients, with 31 of them being girls with CAH and 2 being women with clitoral hypertrophy.

**Conclusion:**

Corporoplasty is a simplified technique for clitoroplasty, with the advantage being that is faster and safer than the technique that involves the dissection of the neurovascular bundle. In addition, corporoplasty has the possible benefit of preserving the cavernosal blood flow that permits the engorgement of the clitoris during sexual arousal.
